# Predisposing Anatomical Patellofemoral Factors for Subsequent Patellar Dislocation

**DOI:** 10.3390/life15081239

**Published:** 2025-08-04

**Authors:** Anna Kupczak, Bartłomiej Wilk, Ewa Tramś, Maciej Liszka, Bartosz Machnio, Aleksandra Jasiniewska, Jerzy Białecki, Rafał Kamiński

**Affiliations:** 1 Faculty of Medicine, Medical University of Warsaw, Żwirki I Wigury 61, 02-091 Warsaw, Poland; 2 Centre of Postgraduate Medical Education, Department of Musculoskeletal Trauma and Orthopaedics, Gruca Orthopaedic and Trauma Teaching Hospital, Konarskiego 13, 05-400 Otwock, Poland

**Keywords:** patellar dislocation, patellofemoral instability, MRI, TT–TG distance, trochlear dysplasia, patella alta, recurrence risk

## Abstract

Background: Primary patellar dislocation is a relatively uncommon knee injury but carries a high risk of recurrence, particularly in young and physically active adolescent individuals. Anatomical features of the patellofemoral joint have been implicated as key contributors to instability. The purpose of this study was to evaluate anatomical risk factors associated with recurrent patellar dislocation following a primary traumatic event, using MRI-based parameters. Methods: Fifty-four patients who sustained a first-time lateral patellar dislocation were included. MRI was used to measure tibial tuberosity–trochlear groove (TT–TG) distance, tibial tuberosity–posterior cruciate ligament (TT–PCL) distance, Insall–Salvati ratio (IS), sulcus angle (SA), patellar tilt angle (PTA), patella length, and patellar tendon length. Trochlear dysplasia was assessed according to the Dejour classification. Recurrence was defined as a subsequent dislocation occurring within three years of the primary injury. Results: Significant differences were observed in TT–TG distance and patellar tendon length (*p* < 0.05). Patients with recurrent dislocation had lower TT–TG values and shorter patellar tendon lengths. Other parameters, including PTA, IS, and patella height, did not show statistically significant differences. Conclusion: Anatomical factors may contribute to the risk of recurrent patellar dislocation. Identifying these variables using imaging may support clinical decision making and guide individualized treatment plans following primary injury.

## 1. Introduction

Patellar dislocation is defined as the displacement of the patella from its normal alignment within the trochlear groove, most commonly in the lateral direction [1,2]. Although it represents only about 3% of all knee injuries, its clinical significance is disproportionate, as an initial dislocation often precipitates recurrent instability [2,3]. The annual incidence of patella dislocation is from 7 to 77 per 100,000 persons [4].

Primary dislocation typically follows a traumatic event and is most frequently observed in adolescents and young adults engaged in athletic activities [5,6,7]. Female patients demonstrate a higher susceptibility than males, with the peak incidence occurring between 10 and 17 years of age [3,8]. Furthermore, a subset of young, physically active individuals possessing certain anatomical knee configurations appears to be particularly predisposed to this injury [6,9]. Despite advances in diagnostic imaging and therapeutic interventions, the risk of recurrence after a first-time dislocation remains substantial, reported in the literature to be between 15% and 60% [7]. To date, few studies have conclusively identified which anatomical or patient-specific factors confer an elevated risk of recurrence. Anatomical causes that may create the risk of such injuries are considered to be patella alta, lateralization of the patella, and trochlear dysplasia [4]. Additionally, the abnormal tracking of the patella could be caused by incorrect soft tissue, bony stabilisers, and joint laxity [4]. The aim of the present study was to characterize the anatomical features of the knee in patients following primary patellar dislocation, with the goal of elucidating the factors that may predispose patients to subsequent dislocations. Identifying the risk factors for subsequent patellar dislocation will allow for better planning of treatment for these patients in the future. It can optimize the patients’ qualification for non-operative or operative treatment after primary injury. The present study addresses several gaps in previous research by incorporating a 3-year follow-up period, analysing a broad spectrum of radiological parameters, analysing the correlation of these parameters, and linking return to activity with various factors. This patient-centered perspective enhances the clinical relevance of the findings.

## 2. Materials and Methods

**Figure 1:**
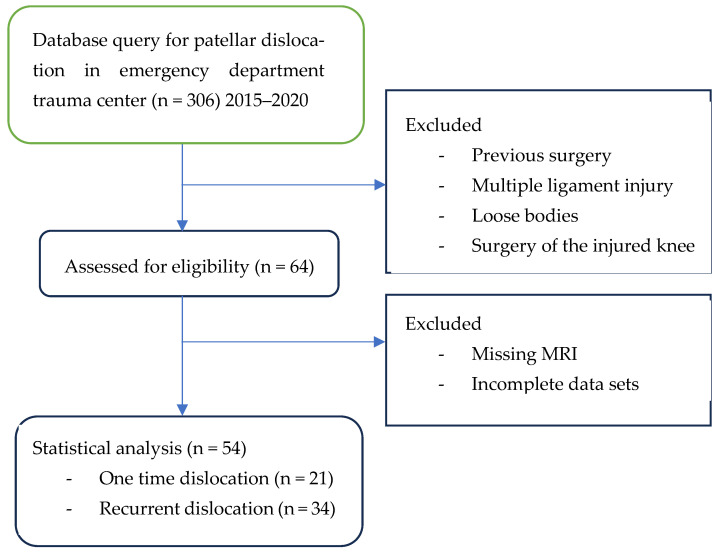
Flow chart.

**Figure 2:**
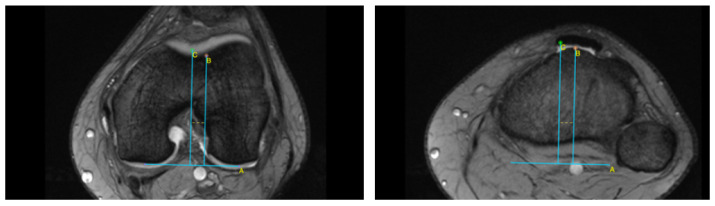
Tibial tuberosity to trochlear groove distance (TT-TG). Line A—posterior condylar axis, line B—most prominent point of tibial tubercle, line C—deepest point of trochlear groove, dotted line—TT-TG.

**Figure 3:**
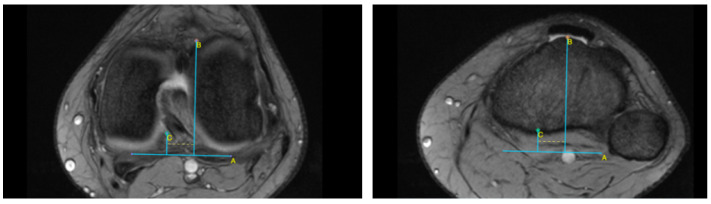
Tibial tuberosity to posterior cruciate ligament distance (TT-PCL). Line A—posterior condylar axis, line B—most prominent point of tibial tubercle, line C—medial border of posterior cruciate ligament, dotted line—TT-PCL.

**Figure 4:**
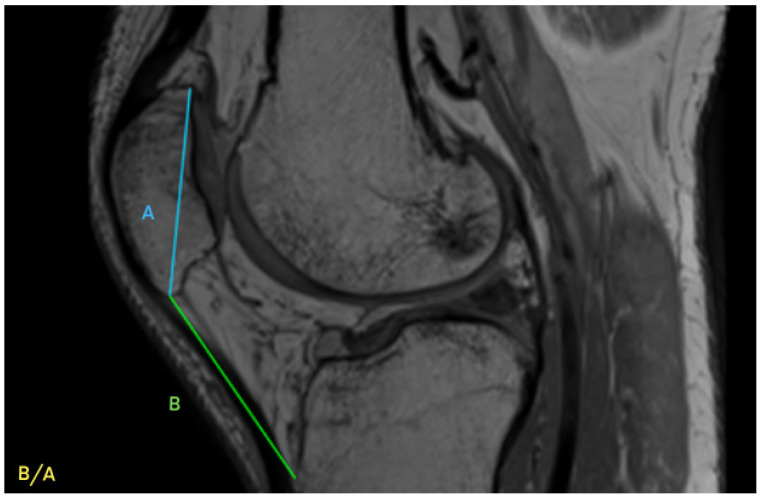
Insall-Salvati index (I-S) with patella length (A) and patellar tendon length (B).

**Figure 5:**
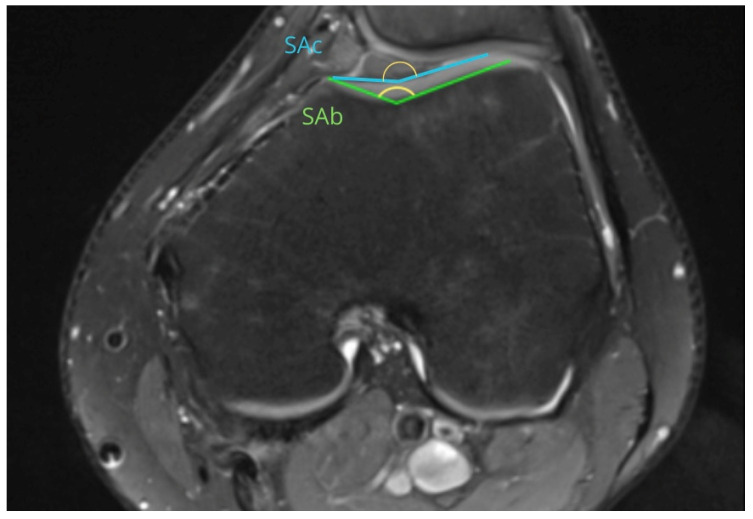
Sulcus angle (SAb—sulcus angle measured on bone, SAc—sulcus angle measured on cartilage).

**Figure 6:**
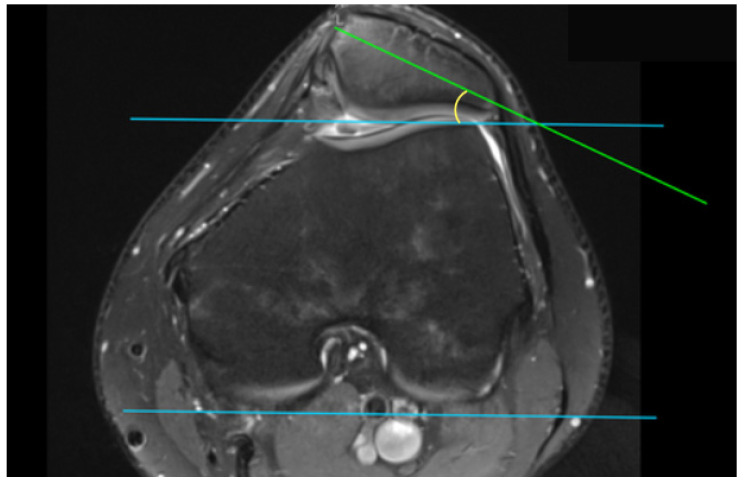
Patellar tilt angle (PTA).

**Figure 7:**
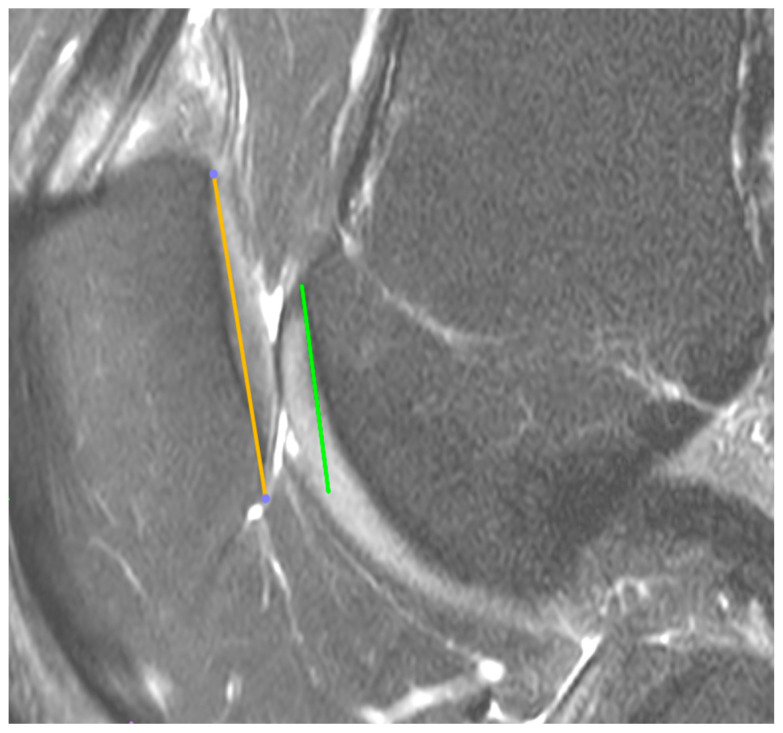
Patella overlap.

This retrospective study was approved by the Institutional Bioethical Committee (no 57/2025). Informed consent was obtained from all patients. Patients were eligible for inclusion in this study if they had suffered acute, first-time patella dislocation. The study cohort comprised 306 patients who sustained patellar dislocation. A total of 252 patients were excluded from this study as they did not meet the inclusion criteria, due to the absence of their MRI data in the hospital computer database, or due to a lack of response in the telephone interview. The final cohort included 54 patients (Figure 1). Patients were recruited from among those who presented to the Emergency Department of the CMKP Gruca Orthopaedic and Trauma Teaching Hospital between 2015 and 2020 due to patellar dislocation. Inclusion criteria were as follows: MRI performed in the hospital during the first follow-up ambulatory visit, non-operative treatment, and the absence of osteochondral loose bodies. Exclusion criteria included a previous surgery on the injured knee, a multiple-ligament injury, and having undergone surgery for the injured knee in the 3 years following the first patella dislocation. MRI evaluations were performed to quantify the following anatomical parameters: tibial tuberosity to trochlear groove distance (TT-TG) (Figure 2), tibial tuberosity to posterior cruciate ligament distance (TT-PCL) (Figure 3), Insall–Salvati index (I-S) with the patella length and patellar tendon length (Figure 4), sulcus angle (SAb—sulcus angle measured on bone; SAc—sulcus angle measured on cartilage) (Figure 5), patellar tilt angle (PTA) (Figure 6), and patella overlap (Figure 7). Additionally, trochlear dysplasia type was assessed using the Dejour classification. An interview was conducted with each selected patient regarding the study procedures. Patients were asked about the year of their first patellar dislocation and whether a subsequent dislocation had occurred within the following three years. They were also asked to provide a subjective assessment of their return to pre-injury knee function during the three years after the initial dislocation. A total of 33 patients reported a recurrent injury. The control group included 21 patients who had suffered from patellar dislocation only once. Patients’ characteristics are presented in Table 1. This study enrolled 54 patients with a mean age of 22.2 years at the time of data collection (range: 10–45 years): 31 women (mean age: 22.2, range: 11–45 years) and 23 men (mean age: 22.2, range: 10–44 years). Their first incident of dislocation occurred at 19.6 years (range: 6–44 years): in women, the first incident occurred at 18.4 years (range: 6–44 years), and in men, the first incident occurred at 20.3 years (range: 6–38 years).

### 2.1. MRI Technique

The MRI was performed on a 1.5-T system, and the knee position was in full extension. The following were obtained: axial and sagittal fat-saturated proton density-weighted turbo spin echo imaging sequences.

### 2.2. MRI Analysis

MRIs were analysed independently by two senior orthopaedic surgeons who had 10 and 5 years of experience in patellofemoral disorders interpretation on MRI and two medical students. All of them were unaware of the results of the previous imaging interpretations. A third senior orthopaedic surgeon reviewed the results for disagreement and reached a consensus. TT-TG distance was assessed on axial MRI images according to Goutallier et al. [10] (Figure 2) and measured between the deepest cartilaginous point of the trochlear groove and the tibial tuberosity at the patellar insertion; additionally, the TT-PCL distance was assessed on axial images as described by Seitlinger et al. [11] (Figure 3), described as the distance between the midpoint of the inferior patellar tendon insertion at the tibial tubercle and the medial border of the posterior cruciate ligament (PCL) at its tibial insertion, measured parallel to the dorsal tibial condylar line; SA was measured as described by van Huyssteen et al. [12] (Figure 5), as the angle between the medial and the lateral aspect of the trochlear groove. The PTA was measured as described by Grelsamer et al. [13] (Figure 6) and is formed between a line referencing the posterior femoral condyles (the rounded ends of the thigh bone at the knee joint) and a line referencing the medial and lateral borders of the patella; and the PO was measured as described by Biedert et al. [14] (Figure 6). PO assesses the extent to which the patella (kneecap) articulates with the trochlear groove of the femur. The IS index was measured on sagittal MRI images as described by Insall et al. [15]; it is a ratio of the patellar tendon length (b) to the patella’s length (A) (Figure 4). The Dejour classification described by Dejour et al. [16] was used to define trochlear dysplasia. The Dejour classification system categorizes trochlear dysplasia into four types (A, B, C, and D) based on the severity and specific features of the dysplasia.

### 2.3. Statistical Analysis

Statistical analysis of acquired data was performed using TIBCO Software Inc. Statistica Version 13.3 for Windows. The Shapiro–Wilk (S-W) test was used to assess the normal distribution of parameters. The Student’s *t*-test and Mann–Whitney U test were used to examine statistical significance. Correlation and regression analyses were conducted using R™ software 4.5.1 (R Foundation for Statistical Computing) [17] with the dplyr package ver. 1.1.3, purr package ver 1.0.2., tidyr package ver. 1.3.0, ggplot2 package ver. 3.5.1, broom package ver. 1.0.5, rstatix package ver. 0.7.2, survival package ver. 3.5–5, and the survminer package ver. 0.5.0. Pairwise linear associations among continuous predictors were assessed using the Pearson correlation coefficient (r), with the strength of relationships interpreted according to conventional thresholds (|r| = 0.00–0.39 = weak, 0.40–0.59 = moderate, 0.60–0.79 = strong, ≥0.80 = very strong). In addition, a generalized linear model (GLM) with a binomial link (logistic regression) was fitted to evaluate the effect of each predictor on the three-year probability of new-onset dislocation. *p* values  <  0.05 were considered to indicate statistical significance.

## 3. Results

A total of 54 patients were included in this study, of whom 21 (39%) were assigned to the control group (only one dislocation within 36 months) and 33 (61%) to the study group (recurrent patella dislocation within 36 months). Overall, 23 patients (43%) successfully returned to their pre-injury level of activity, whereas 31 (57%) did not. In the study group, 10 patients (30%) resumed participation in sports activities, compared with 13 patients (62%) in the control group. Conversely, 23 patients (70%) with recurrent dislocations and only eight patients (38%) with one-time dislocations did not return to their pre-injury level of activity. The cohort consisted of 31 women and 23 men, with a female predominance in both groups—18 (55%) in the recurrent group and 13 (62%) in the control group. Baseline characteristics are presented in Table 2.

### 3.1. Subsequent Dislocation

A statistically significant difference in the patellar tendon length was observed between the two groups (*p* < 0.05). Patients in the subsequent dislocation group had a significantly shorter patellar tendon length (54.15 ± 6.91) compared to the control group (59.14 ± 5.88). No statistically significant differences were found between the groups with regard to TT-PCL, SAb, SAc, patellar length, and overlap, and the I-S ratio between the groups was not statistically significant. The results are presented in Table 3.

A statistically significant difference in the TT–TG distance was observed between the groups (*p* < 0.05). Patients in the subsequent dislocation group had a significantly lower TT-TG distance (10.12 ± 4.46) than the control group (12.29 ± 4.63). The differences in age and PTA between the groups were not statistically significant. The results are presented in Table 4.

### 3.2. Return to Activity (In the Whole Study Population)

The differences in TT-PCL, SAb, SAc, patellar tendon length, and the I-S ratio between the groups were not statistically significant. The results are presented in Table 5.

The differences in age, TT-TG, PTA, and patella length between the groups were not statistically significant. The results are presented in Table 6.

### 3.3. Dejour Dysplasia

Dejour classification did not show a statistically significant difference among groups; however, in groups B, C, and D, twice as many patients had that type of dysplasia, and in Group A, more patients with recurrence had this mild type. (Table 7).

### 3.4. Age

**Figure 8:**
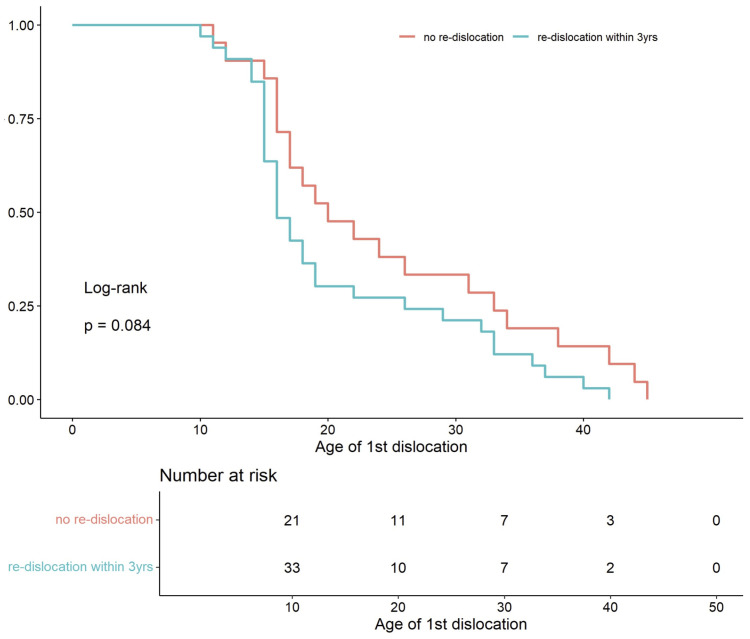
Kaplan–Meier curves for time to first patellar dislocation, comparing patients with recurrence (blue line) versus those without recurrence (red line) (log-rank *p* = 0.08).

Interestingly, patients with recurrent dislocation were younger at the time of first dislocation (median 16) compared to those without another dislocation (median of 20). The survival curves (Figure 8) illustrate this trend; however, the difference did not reach statistical significance.

### 3.5. Correlation

**Figure 9:**
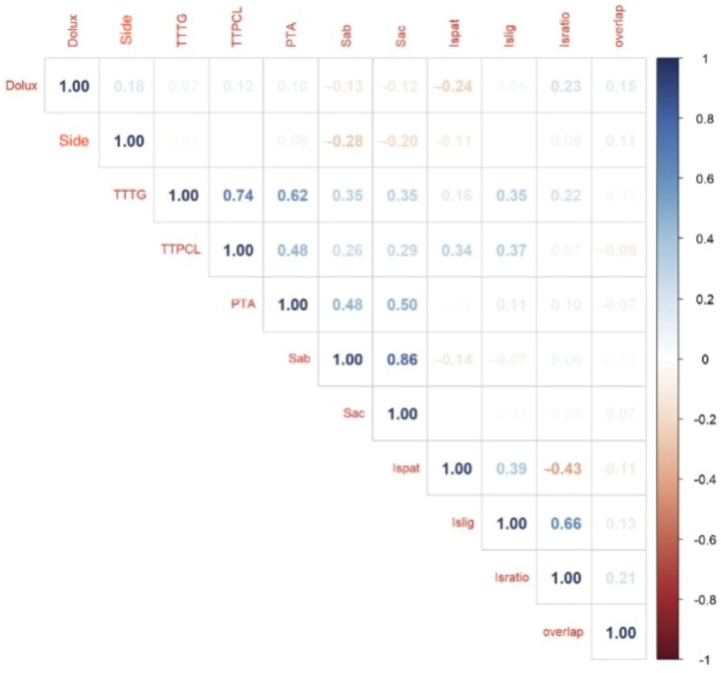
Correlation; side—left/right, Dolux—Date of luxation TTTG—tibial tuberosity trochlear groove, TTPCL tibial tuberosity posterior cruciate ligament, PTA—patella tilt angle, Sab—sulcus angle bone, Sac—sulcus angle cartilage, Ispat—IS patella length, ISlig—patellar tendon length, ISratio—Insall-Salvati ratio, overlap—patella overlap.

The results of the correlation analyses of the measured factors are shown in Figure 9. A strong positive correlation was found between both SA measurements (0.86) and TT-TG and TT-PCL (0.74).

### 3.6. Interaction Effects on Recurrence Risk

Table 8 reports the adjusted odds ratios (OR), 95% confidence intervals (CI), and *p*-values for both the main effect of primary factors and their significant interactions from the linear and multivariable logistic regression model. Returning to the previous level of activity within three years was independently associated with a 73% reduction in the odds of recurrent dislocation (OR 0.27, 95% CI 0.08–0.83; *p* = 0.0249). However, this effect was influenced by patellofemoral anatomy. A greater patella length attenuated the protective benefit, such that each millimetre increment was associated with a 14% increase in recurrence odds (OR 1.14, 95% CI 1.03–1.29; *p* = 0.016). Moreover, the combination of increased patellar tilt angle and trochlear overlap synergistically amplified risk nearly 100-fold (OR 97.40, 95% CI 4.34–2.46 × 10^4^; *p* = 0.021). When patellar ligament length was also included, this three-way interaction conferred an additional 12% rise in odds (OR 1.12, 95% CI 1.04–1.29; *p* = 0.0257). These data indicate that resuming full activity is beneficial; its protective value depends on underlying patellofemoral morphology, particularly the dimensions and alignment of the patellofemoral joint.

## 4. Discussion

Anatomical factors have been extensively studied to understand their pivotal role in recurrent patellar dislocations. Trochlear dysplasia, marked by a shallow or flattened trochlear groove, has been shown to increase the odds of recurrence (OR 4.15, *p* = 0.009). Patella alta, in which the patella is positioned higher than normal, carries an OR of 2.38 (*p* = 0.004) for recurrent dislocations. Likewise, an elevated TT–TG distance, reflecting lateralization of the patellar tendon attachment, is associated with recurrence (OR 2.87; *p* < 0.00001) [18]. Other anatomical factors affecting the first-time dislocation include trochlear dysplasia, patellar tilt, patella alta, tibial tuberosity to trochlear groove distance, and trochlear groove depth [19,20]. These findings have been corroborated by additional studies [4,21,22,23].

Interestingly, in contrast to most previous research, including that cited above, which identified increased TT-TG distance as a risk factor for recurrent patellar dislocation, our results suggest the opposite. Patients with a reduced TT-TG distance appeared to have a statistically higher risk of recurrent dislocation. This unexpected finding is particularly intriguing. Potential explanation includes the presence of other significant risk factors, such as soft tissue insufficiency like quadriceps imbalance, which increases the risk of injury, despite the absence of differences in the parameters we measured. Methodological differences and sample-specific characteristics that may have influenced the outcome should also be considered. Nevertheless, this finding challenges the conventional understanding of patellofemoral instability and opens new avenues for further research into patient-specific risk factors beyond isolated radiographic parameters.

Traumatic patella dislocation typically results in injury to the medial stabilizing structures of the knee, notably the medial patellofemoral ligament (MPFL) [5]. The severity of MPFL rupture, along with the presence of osteochondral lesions, influences both the prognosis and the choice of intervention [24]. There is an ongoing debate regarding the role of surgical intervention following a primary patellar dislocation. Current clinical practice generally reserves surgical procedures—such as MPFL reconstruction, trochleoplasty, or tibial tuberosity transfer—for patients with recurrent dislocations, significant anatomical abnormalities, or patellar subluxation [25,26].

In addition to established anatomical risk factors, dynamic contributors, namely muscular imbalance and patellar maltracking, have been implicated in recurrent patellar dislocation. Weakness or dyscoordination of the quadriceps, particularly the vastus medialis oblique, predisposes patients to abnormal patellar tracking, thereby increasing the likelihood of dislocation, especially when combined with other anatomical risk factors [27]. Abnormal femoral torsion, particularly excessive femoral anteversion, further disrupts patellar mechanics and contributes to recurrence [28]. These findings—including MPFL rupture and muscle imbalances—may also explain why patients with otherwise normal patellofemoral anatomy remain at high risk for recurrent patellar dislocation.

An interesting finding is that returning to physical activity has a protective effect against recurrent dislocations. Although functional recovery is generally associated with improved dynamic stability of the patellofemoral joint, our results indicate that this benefit is not absolute and may be highly dependent on underlying anatomical factors. Specifically, certain morphological characteristics may reduce or even nullify the stabilizing effect of muscle control and coordination restored through rehabilitation. The finding that the protective effect of resuming activity is reduced by unfavorable patellar morphology, such as increased patellar length or inclination, suggests that unfavorable biomechanical factors may be more important than neuromuscular recovery in some patients. For instance, an elongated patella may impair congruence with the trochlear groove or unfavorably alter force vectors during movement, thereby limiting the efficacy of dynamic stabilization. Similarly, structural abnormalities, such as increased inclination or trochlea overlap, may indicate deeper soft tissue imbalances or abnormal movement patterns that are not easily corrected by muscle action alone. These results emphasize the need for a more nuanced approach to return-to-sport recommendations. Rather than assuming physical activity to be generally beneficial, clinicians should consider how individual morphological profiles may alter its effects. This points to a broader principle in the treatment of patellofemoral instability: anatomical and dynamic factors interact in a complex manner, and optimal treatment strategies should reflect this interaction. Future research should focus on identifying which combinations of static and dynamic risk factors most reliably predict recurrence and how rehabilitation protocols can be tailored accordingly.

Understanding these anatomical predispositions enables more precise selection between conservative and surgical management. Conservative management consisting of initial immobilization followed by structured rehabilitation remains the standard approach after a first-time dislocation, particularly when anatomical risk factors are mild and no patellar subluxation is observed [29,30]. Surgical intervention may be considered for patients with significant anatomical abnormalities that predispose them to recurrence [23,31].

Patients with pronounced trochlear dysplasia or patella alta may benefit from surgical correction, such as trochleoplasty, tibial tuberosity transfer, or medial patellofemoral ligament reconstruction, to restore patellar stability [30]. Early identification of risk factors through advanced imaging and thorough clinical assessment informs both surgical decision-making and the design of rehabilitation protocols. Tailored rehabilitation, targeting quadriceps imbalances, optimizing patellar tracking, and enhancing muscle strength can markedly reduce recurrence, especially when combined with individualized activity and sport participation guidelines [32]. Finally, the development and implementation of a standardized risk-stratification tool, based on these anatomical variables, could further refine patient selection and optimize treatment strategies across clinical settings.

## 5. Strengths and Limitations

While several strengths are evident, certain limitations are outlined below.

All anatomical measurements were obtained using MRI, ensuring a high degree of accuracy in evaluating key parameters such as TT–TG distance, PTA, and trochlear dysplasia type. The inclusion of a control group of patients who did not experience recurrent dislocation allows for more meaningful interpretation of risk factors. Additionally, the 3-year follow-up period strengthens the reliability of the data regarding the recurrence of patellar dislocation after primary injury.

The relatively small cohort (n = 54) may reduce the statistical power of the findings. The retrospective character of this study did not allow for the collection of data such as patient-reported outcomes at the time of dislocation. Details regarding rehabilitation protocols were not included, yet these factors could have influenced the likelihood of successful recovery or recurrence. Only static parameters were measured, while rotational alignment and lower limb axis were not assessed in this study.

## 6. Conclusions

Anatomical factors that significantly predispose patients to recurrent patellar dislocation include a decreased tibial tuberosity–trochlear groove (TTTG) distance and shortened patellar tendon length. Age at the time of initial injury also emerged as an important risk modifier. Notably, returning to the pre-injury level of activity within three years appeared to have a protective effect against recurrence. Although other parameters, commonly reported as elevated in previous studies, were evaluated, they did not reach statistical significance in this cohort. Further research is therefore necessary to fully assess their potential role.

Identifying these key risk factors is crucial for making informed decisions regarding the need for surgical intervention following a primary dislocation. The findings suggest that TT–TG distance and patellar tendon length should be considered important criteria when evaluating patients for early surgical treatment.

By systematically identifying and analyzing factors predisposing to subsequent dislocation, this study aims to contribute to the development of individualized risk-assessment tools for patients following a first-time injury [23]. Such tools are essential for guiding clinical decision-making, enabling healthcare providers to tailor treatment plans that effectively mitigate recurrence. This targeted approach has the potential to improve outcomes and reduce the long-term complications resulting from patellar instability. Incorporating these risk factors into surgical indication protocols has been associated with lower re-injury rates [23,33,34]. Specifically, while conservative management may be sufficient for low-risk patients, those with pronounced anatomical predispositions may benefit from early surgical intervention to prevent recurrence.

## Figures and Tables

**Table 1:** Demographic and clinical characteristics.

Characteristics	Patients
Sex:	
Female Male	31 23
Age at the time of first dislocation	19.2 (6–44)
Mean age	22.2 (6–44)
Right knee	23
Left knee	31
Return to activity: Primary dislocation Recurrent dislocation	13 (62%) 10 (18%)

**Table 2:** Baseline characteristics of the control and study groups.

	Control Group	Study Group
Patients	21 (39%)	33 (61%)
Female	18 (55%)	13 (62%)
No return to activity	8 (38%)	23 (70%)
Return to sport	13 (62%)	10 (30%)

**Table 3:** Differences between the subsequent dislocation group and the control group in the parameters with normal distribution in both groups.

	Subsequent Dislocation	Control Group	*p*
TTPCL	20.88 ± 5.46	23.24 ± 4.81	0.11
SAb	148.26 ± 13.82	149.93 ± 10.66	0.64
SAc	156.06 ± 12.43	159.01 ± 10.69	0.37
Patella length	39.42 ± 3.74	41.86 ± 5.41	0.06
Patellar tendon length	54.15 ± 6.91	59.14 ± 5.88	0.008 *
I-S ratio	1.38 ± 0.17	1.43 ± 0.18	0.32
overlap	0.31 ± 0.1	0.28 ± 0.05	0.3

*p* for the control group vs. subsequent dislocations group; Data are presented as mean ± standard deviation unless otherwise indicated. * Statistical significance was defined as *p* < 0.05 (student’s *t* test).

**Table 4:** Differences between the subsequent dislocation group and the control group in the parameters with non-normal distribution in at least one group.

	TTTG	PTA	Age
Subsequent dislocation group	10.12 ± 4.46	20.74 ± 10.84	17.21 ± 7.76
Control group	12.29 ± 4.63	22.64 ± 7.61	22.42 ± 11.36
U-Test *p*	0.046	0.41	0.09

*p* for the control group vs. subsequent dislocations group; Data are presented as mean ± standard deviation unless otherwise indicated. Statistical significance was defined as *p* < 0.05 (U-test).

**Table 5:** Differences between the successful return to activity group and the unsuccessful return to activity group in the parameters with normal distribution in both groups.

	TTPCL	SAb	SAc	Patellar Tendon Length	I-S Ratio
Successful return to activity group	21.41 ± 4.85	150.60 ± 10.78	158.62 ± 10.85	55.23 ± 7.37	1.39 ± 0.19
Unsuccessful return to activity group	22.30 ± 5.66	146.62 ± 13.72	155.30 ± 12.38	57.26 ± 6.55	1.41 ± 0.16
*t*-Test *p*	0.55	0.26	0.31	0.29	0.56

*p* for the control group vs. subsequent dislocations group; Data are presented as mean ± standard deviation unless otherwise indicated. Statistical significance was defined as *p* < 0.05 (student’s *t* test).

**Table 6:** Differences between the successful return to activity group and the unsuccessful return to activity group in the parameters with non-normal distribution in at least one group.

	Age	TTTG	PTA	Patella Length
Successful return to activity group	19.29 ± 9.81	10.87 ± 4.54	21.63 ± 8.3	40.06 ± 4.8
Unsuccessful return to activity group	19.17 ± 9.56	11.09 ± 4.72	21.29 ± 10.72	40.78 ± 4.46
U-Test *p*	1.0	0.68	0.93	0.85

*p* for the control group vs. subsequent dislocations group; Data are presented as mean ± standard deviation unless otherwise indicated. Statistical significance was defined as *p* < 0.05 (U-test).

**Table 7:** Distribution of Dejour dysplasia groups.

Dejour Type	No Recurrence	Recurrence	Total
A	9	6	15
B	3	5	8
C	6	11	17
D	2	4	6

**Table 8:** Adjusted odds ratios (OR), 95% confidence intervals (CI) and *p*-values for both the main effect of primary factors and its significant interactions from the linear and multivariable logistic regression model.

Factor	OR	95% CI	*p*-Value
return to previous level of activity	0.27	0.081–0.826	0.0249
patella length return to previous level of activity	1.1389	1.0345–1.2879	0.01649
PTA Overlap	97.398	4.344–2.46 × 10^4^	0.02126
PTA patella ligament length overlap	1.1159	1.0406–1.2855	0.02569

## Data Availability

Dataset available on request from the authors.

## Abbreviations

The following abbreviations are used in this manuscript:

MRI	Magnetic resonance Imaging.
TT–TG	tibial tuberosity–trochlear groove
TT–PCL	tibial tuberosity–posterior cruciate ligament
IS	Insall–Salvati ratio
SA	Sulcus angle
PTA	patellar tilt angle
